# An Empirical Framework for Assessing the Balanced Scorecard Impact on Sustainable Development in Healthcare Performance Measurement

**DOI:** 10.3390/ijerph192215155

**Published:** 2022-11-17

**Authors:** Anca Antoaneta Vărzaru

**Affiliations:** Department of Economics, Accounting and International Business, University of Craiova, 200585 Craiova, Romania; anca.varzaru@edu.ucv.ro; Tel.: +40-7-7392-1189

**Keywords:** balanced scorecard, digital transformation, accounting information system, strategic human resources management, sustainable development, efficiency, effectiveness

## Abstract

Performance appraisal has become an essential tool for healthcare managers due to the frequent and rapid changes in the healthcare sector. Sustainable performance management implies increasing organizations’ efficiency and effectiveness while considering all vectors of sustainability. This study examines the impact of digital transformation, accounting information systems, and strategic human resource management improvements on organizational performance and sustainable development. The paper uses the balanced scorecard (BSC) for organizational performance assessment. The paper proposes a theoretical model that integrates the traditional and digital information systems and human resources engagement with the BSC dimensions for sustainable organizational development. The theoretical model is tested in an empirical study involving a questionnaire-based survey of 387 employees with management experience in the healthcare sector. Based on structural equation modeling, the research results showed that the efficiency and effectiveness of strategic human resources management and the accounting information system significantly positively affect the BSC dimensions. Furthermore, the use of BSC in measuring organizational performance has significant effects on sustainable development, with the internal process dimension being the most influential.

## 1. Introduction

The trend toward sustainable development causes many organizations in the healthcare sector to choose performance evaluation systems that capture all dimensions of performance [1]. Patients and other stakeholders pay more attention to evaluating sustainable performance, given that the economy based on knowledge and digitization creates value, primarily through intangible assets, which are extremely difficult to evaluate. Until the 1990s, organizations used performance evaluation methods based only on financial accounting data and using only financial criteria. Intangible (intellectual) assets generate more added value and significantly contribute to competitive advantage, being unquantifiable from a financial perspective. Intangible assets represent the knowledge and skills of human resources, the capabilities of accounting information systems, and the innovative features of digital technologies used in organizational activities. The significant advantage of intangible assets lies in the difficulty of imitation by similar organizations. The main disadvantage consists of the difficulty of the performance evaluation of intangible assets [2]. Traditional methods, oriented toward financial measures, did not provide information that would allow solving problems or capitalizing on opportunities related to customers, quality, and personnel [3]. Therefore, multiple opinions appeared in performance evaluation regarding the need to measure intangible assets using non-financial criteria [4].

As a result of this trend, organizations have chosen to adopt multidimensional approaches to performance evaluation in line with organizational strategy [5]. Organizations evaluate actual performance using multidimensional performance evaluation methods suitable to the structure and diversity of assets [6]. Since the 1990s, organizations have implemented various multidimensional performance assessment methods considering intangible (intellectual) assets: Performance Prism, Performance Pyramid, Skandia Navigator, Performance Measurement Matrix Model, and Balanced Scorecard (BSC).

The BSC allows the translation of an organization’s strategic objectives into performance objectives. Within the BSC, the evaluation of organizational performance has four dimensions: the financial dimension (FD), the customer dimension (CD), the internal process dimension (IPD), and the learning and growth dimension (LGD) [7]. Later, various authors found that the BSC can be used not only as a performance evaluation method but also as a strategic management model [8,9].

Organizations increasingly use the Balanced Scorecard in the healthcare sector due to increasing complexity and the need to implement efficient management systems to measure performance [10,11,12,13,14,15,16,17,18,19].

Digital transformation has facilitated using of a complex tool such as the Balanced Scorecard. Although digital technologies have expanded a lot, especially in the last three years in the context of the COVID-19 pandemic, the potential of digital technologies remains significant and is not sufficiently valued [20]. This potential must be exploited through the involvement and engagement of human resources in healthcare, particularly young professionals [16,20]. Only strategic human resource management organizations achieve a complete integration of digital strategies with human resources strategies [16]. A challenge for effectively implementing a digitized Balanced Scorecard is the interoperability of traditional and digital information systems [20,21,22].

The framework proposed by Palozzi et al. [23], Health Technology Balanced Assessment, integrates BSC with Health Technology Assessment (HTA) to foster hospital-based health technology management to align strategy and action. However, no clear instrumental framework allows an integrated implementation of BSC with HTA. The model proposed in the paper provides the necessary tools to integrate the BSC with the HTA, analyzing the relationships between digital transformation, accounting information systems, strategic human resources management, and BSC dimensions. The instrumental framework for BSC optimization is a gap found in the literature concerning healthcare, which the paper addresses through a proposed theoretical model tested in the empirical study. Therefore, the paper aims to examine the direct impact of digital transformation, the improvement of the accounting information system, and the strategic human resources management on the organizational BSC performance dimensions and the indirect impact on the sustainable development of organizations in the healthcare sector. By examining these causal relationships, the paper strengthens the role of BSC in sustainable development, having as input vectors the digital transformation, the improvement of the accounting information system, and the strategic human resources management optimization.

The findings provide hospital managers with the necessary tools for sustainable development through digital and informational transformation and the engagement of human resources in strategic processes.

The paper consists of six sections. The Section 2 carries out a literature review. In Section 3, the paper highlights the research design and methodology. Section 4 reveals the results after the analysis and interpretation of the collected data, and Section 5 and Section 6 include the discussions, theoretical and managerial implications, research limitations, and conclusions.

## 2. Theoretical Background

### 2.1. Performance Assessment Using BSC

Performance evaluation quantifies production factors’ contribution to organizational performance [24]. The main reason for performance evaluation is to increase performance concerning the previous period and similar organizations [25]. In addition, psychological research on performance appraisal has emphasized performance improvement through transformational feedback, which focuses on developing organizational capacity to perform [26].

According to Kaplan and Norton [5], BSC is a management tool that helps an organization operationalize its strategy. The BSC translates the vision and strategy of an organization into performance indicators from a strategic measurement and management system. BSC represents a valuable tool that facilitates the strategies’ implementation and the development of strategic objectives [27]. The BSC creates an equilibrium between traditional financial and non-financial indicators: customers, the organization’s internal processes, and learning and organizational development processes. Since the BSC includes financial and non-financial variables, its approach is balanced. BSC dimensions contribute to organizational operations with strategic objectives. Neely and Bourne [28] show that BSC success depends on how the indicators are defined and implemented. Chaudron [29] believes that BSC balances long-term and short-term objectives. Most employees are unaware of the organizational strategy and objectives; therefore, they do not understand their role in achieving the organization’s purpose. As a result, employees strictly fulfill their assigned individual goals [30] due to the absence of cooperation among employees in achieving the organization’s strategies and goals. Moreover, many employee reward systems focus on individual or team success rather than on achieving organizational goals [31].

According to Frigo and Krumwiede [32], BSC can contribute to better strategic human resources management because it ensures the interconnection between personal and organizational objectives and assesses the effects on organizational performance. The organization can achieve long-term objectives by converting the vision and strategy into individual objectives and quantitative and qualitative indicators, generating an optimal framework for communicating the vision and strategy to all employees [5,9,33].

As a result of strategic performance evaluation capabilities, BSC has a crucial role in strategy analysis and implementation and in assessing organizational goals [27,34,35,36,37,38,39]. In the last two decades, various researchers addressed the implementation of BSC by leading companies in various sectors [38], including the public sector [35,40,41] or the healthcare sector [14,16,19].

### 2.2. Digital Transformation

The use of digital technologies and innovations in healthcare organizations significantly impacts organizational performance [9] due to increasing accessibility, speed, transparency, innovativeness, and trust. The level of implementation of digital technologies defines the level of digital transformation (DT) of activities and significantly affects the decision-making process by increasing the speed of decision-making and reducing errors [42,43]. In addition, many organizations integrate new digital technologies into the accounting information system used by managerial accounting [44,45,46,47,48,49,50,51,52,53]. Therefore, accounting management tools know an increasingly accelerated digital transformation [54,55,56,57,58,59,60,61,62,63,64,65].

DT consists of implementing new digital technologies in a business model, improving organizational processes, redesigning the value creation chain, and better satisfying customer needs [27,66,67]. Formulating a strategy in the DT field is not enough to successfully implement digital transformations because the implementation of digital technologies and their strategic integration is a broader concept [27,68]. However, since DT is an organizational transformation much more than digital technologies, the success of DT depends on the integration into organizational processes and the acceptance of these technologies by employees. Consequently, DT initiatives must be integrated into accounting information systems and aligned with strategic human resource management to fully integrate into an organization’s strategy.

BSC essentially represents a new organizational approach involving new ways of thinking, innovation, and organizational change. Various researchers [69,70] analyzed the relationship between DT and the application of BSC at the level of various organizations, while Yamamoto [71] and Zanon et al. [72] studied BSC use as a potential method for DT. DT provides the BSC with accuracy and easier use, given the complexity of the indicators included in the BSC [73,74]. Implementing IT solutions based on artificial intelligence, Big Data, and the Internet of Things can address this complexity. Digital BSC can make managers’ work more accessible. Data (Big Data) collected by information systems and physical sensors of the Internet of Things technology are then processed and interpreted with the help of artificial intelligence capabilities. Artificial intelligence offers the possibility of quick repetitive decisions without human intervention and facilitates the adoption of strategic decisions.

The BSC model aims to reduce the gap between strategy and implementation and eliminate redundant activities. Consequently, implementing a strategy in the field of DT would be supported by using BSC. DT positively influences BSC dimensions, particularly FD [27,68,70]. The BSC implementation can be made much easier with the help of digital technologies integrated into the organization’s strategy and the performance evaluation system [23].

Monitoring the DT effects is very important to assess the change produced within the organization, be it positive or negative, and the influences on performance and sustainable development. Tracking these changes with the help of the BSC facilitates the analysis of the actual situation, not just some financial indicators, the earlier identification of the operational problems, and the faster finding of solutions to solve the problems. Therefore, the indicators used to evaluate innovative activities must be quantifiable, reliable, simple, and meaningful because the decisions depend on these indicators [75,76]. Furthermore, innovative organizations encourage experimentation, reward successes, and actively support their employees’ training and growth [73,74,77,78,79].

Previous research [27,70,71,72,73,74,77,78,79] shows a positive relationship between innovation achieved through DT and BSC dimensions. On this basis, the first hypothesis is the following:

**Hypothesis** **H1.** *DT has a significant positive effect on BSC dimensions.*

### 2.3. Accounting Information System

A healthcare organization’s accounting information system (AIS) collects and processes data and provides financial and operational information. AIS mixes accounting, financial and managerial approaches with the capabilities offered by software specialized in managing organizational information [80].

The advantages of the AIS efficiency and effectiveness are the following: improvement of the quality, quantity, and speed of information circulation increased adaptability to a constantly changing economic environment, operational management improvement, communication channel optimization, and increased opportunities regarding external relationships. As a result of increasing informational capabilities, organizations have more opportunities for diversification [81,82,83]. According to Ditkaew [84], the AIS quality significantly influences business performance.

Verboncu and Zalman [85] show that performance measures of organizational efficiency and effectiveness; these concepts can measure BSC dimensions. Therefore, responsible accounting information systems should measure performance in all aspects, not only the financial aspect. Thus, organizations’ accounting information systems can improve in the long term to evaluate non-financial performance, contributing to the organization’s sustainable development [85]. The continuous adaptation of accounting information systems to technological modifications leads to a workplace redesign. The role of professionals translates from data collection and process activities to complex decision-making tasks, problem-solving, and tactical communication with internal and external stakeholders [86]. On this basis, the second hypothesis is the following:

**Hypothesis** **H2.** *The AIS improvement has a significant positive effect on the BSC dimensions.*

### 2.4. Strategic Human Resources Management

In the contemporary economy, and even more so in organizations in the healthcare sector, human capital represents an intellectual capital consisting of knowledge, skills, and competencies through which an organization can obtain a competitive advantage [33,87].

Strategic human resources management (SHRM) primarily influences the learning and development dimension within the BSC and the internal process dimension of a firm’s organizational performance [32,88]. Findikli et al. [89] and Amer et al. [90] show that SHRM can improve ‘employees’ motivation by ensuring performance increases. In addition, human resource strategies significantly positively impact organizational performance [91], affecting the organization’s customer relations and financial results [92,93]. Therefore, human resource is an essential and valuable asset for every organization, difficult to replicate or replace, contributing to sustainable performance.

The potential of digital technologies must be harnessed through the involvement and engagement of human resources in healthcare, particularly of young professionals who are more open to using technologies in any activity [16,20,90]. Furthermore, only through an SHRM can organizations integrate digital strategies with human resources strategies and contribute to optimal BSC implementation [90,93].

Amer et al. [90] consider that the engagement of healthcare staff in implementing BSC could solve the problem of reluctance regarding digital transformation, increasing the satisfaction levels of employees. At the same time, Amer et al. [90] find that the involvement of health personnel in BSC implementation will improve all BSC dimensions.

On this basis, the third hypothesis is the following:

**Hypothesis** **H3.** *The SHRM improvement significantly positively affects the BSC dimensions.*

### 2.5. Impact of BSC on Sustainable Development

Financial and non-financial performance is essential for every organization regarding sustainable development objectives. The model of organizational transformation through digital transformation must also include the objectives of sustainable development [27]. To evaluate non-financial performance as part of sustainable performance, the BSC is a valuable tool for improving the sustainable performance of organizations [94]. The BSC facilitates organizations’ growth and sustainable development [95,96]. Recent studies have shown a high integration between the balanced scorecard and sustainable development [96]. Non-financial indicators are motivating factors to enhance sustainable development regarding social and environmental issues [97]. Learning and growth are crucial elements for organizational growth and development [98]. The adoption of the BSC is essential for ensuring the organization’s sustainable development because it uses financial indicators that characterize the economic driver and operational indicators that characterize the social and environmental drivers. For better BSC use, synchronizing sustainable development and strategic management systems is fundamental [96].

Butler et al. [99] and Kalender and Vayvay [100] suggested the development of a framework for a sustainable BSC. The new model offers an additional perspective that encompasses economic, social, and environmental aspects. In such a model, social and environmental drivers fit into all BSC perspectives. However, in the corporate world, environmental and social goals often conflict with financial goals [101]. These contradictions reflect in the development of a sustainable BSC model. However, social and environmental goals ultimately lead to financial goals.

To focus on sustainable development, managers in the healthcare sector must benefit from appropriate mechanisms [102]. In addition, many researchers have indicated the benefits of BSC for the organization’s sustainable development, obtaining a competitive advantage over other organizations [103,104]. On this basis, the fourth hypothesis is the following:

**Hypothesis** **H4.** *BSC dimensions have a significant positive effect on sustainable development.*

**Figure 1:**
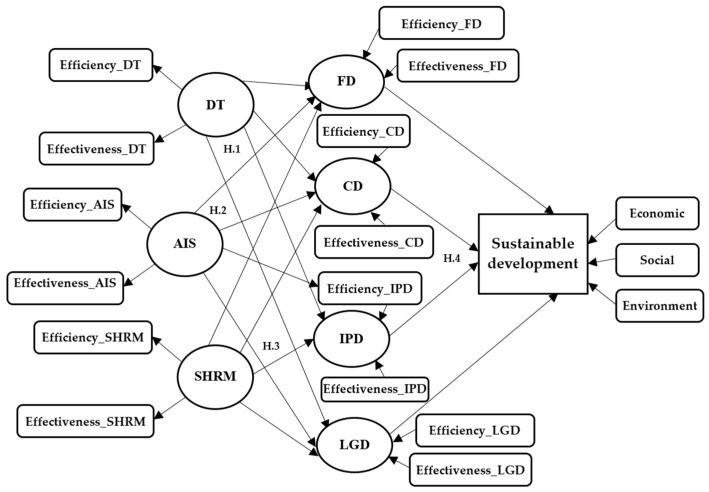
Theoretical model. Source: own construction based on [7,9,31,42,106,107].

According to Kilig and Uludag [105], the primary strategic objective of organizations is to improve performance by increasing efficiency (the ability to achieve set objectives with minimum resources) and effectiveness (achievement of the established objectives). Organizations’ performance levels are consistent with current technologies, information, human resource strategies, and performance evaluation methods. Figure 1 shows the research model, the relationships between the research variables, and the research hypotheses.

## 3. Research Design and Methodology

**Figure 2:**
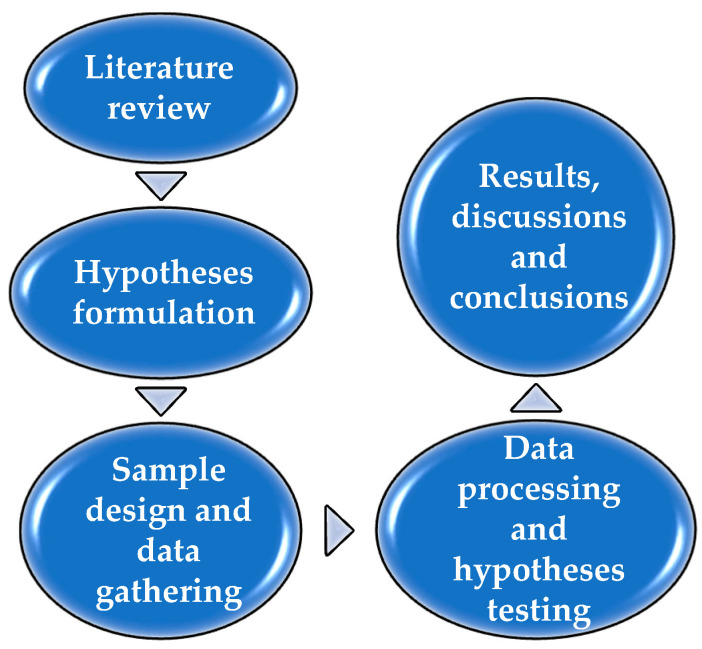
Research process phases. Source: own construction.

Investigating the direct impact of DT, the improvement of the AIS and SHRM on BSC dimensions, and the indirect impact on sustainable development in the perception of employees with management experience in the healthcare sector involved five phases (Figure 2).

The study used a survey-based approach with a questionnaire to assess the ‘employees’ perception of management experience in the healthcare sector. The study used the stratified random sampling method. The establishment of the layers depended on two demographic criteria (gender and age). The study population consisted of Romanian employees with experience in management positions in the healthcare sector. The survey took place in the South-West Oltenia region. The respondents were among the employees with experience in top management and middle management positions (managers of medical departments, auxiliary departments, financial offices, and accounting offices). The sample has a level of confidence of 95%, with the margin of error being 4.565%. The questionnaire was sent to 500 people to ensure a suitable sample with a high confidence level and a small margin of error. Out of the 500 questionnaires sent, 398 respondents returned the filled questionnaires. Therefore, 387 questionnaires are valid (duly filled). The response rate was 75.60%. The questionnaire was distributed by email between June 2022 and September 2022. Among the total respondents, 54.3% are male, and 45.7% are female. The structure according to age is as follows: 25.1% of respondents are in the 18–30 years category, 43.1% are in the 31–45 years category, and 31.8% of respondents are between 46 and 65 years.

The questionnaire consists of nineteen questions (Table A1). The questionnaire items were developed based on previous research on balanced scorecard dimensions, digital transformation, accounting information systems, human resources strategic management, and sustainable development [7,9,31,42,106,107]. Two questions in the first part of the questionnaire contained demographic information. The following sections include the antecedent variables of digital transformation, accounting information system improvement, strategic human resource management, BSC dimensions, and sustainable development (Table 1).

The questions for the antecedent variables of DT, the improvement of AIS and SHRM, and the BSC dimensions are as follows: “On a scale from 1 to 5 (1—non-important, 5—most important) what do you think is the importance of [DT/AIS/SHRM/FD/CD/IPD/LGD] in increasing organizational [efficiency/effectiveness]”. The questions for the antecedent variables of sustainable development are as follows: “On a scale of 1 to 5 (1—non-important, 5—most important), what do you think is the importance of [economic driver/social driver/environmental driver] in ensuring sustainable development”. The questionnaire includes general questions regarding the employees’ perceptions and does not include data that require an institutional review board and informed consent. The measurement scales were developed based on previous research [7,9,31,42,106,107]. DT, AIS, SHRM, FD, CD, IPD, LGD, and sustainable development are the model’s endogenous (latent) variables. Table 2 shows the descriptive statistics of the observable variables (questionnaire items).

The paper used structural equation modeling to test research hypotheses. Structural equation modeling allows the assessment of the relationships among the model’s latent variables [108].

## 4. Results

**Figure 3:**
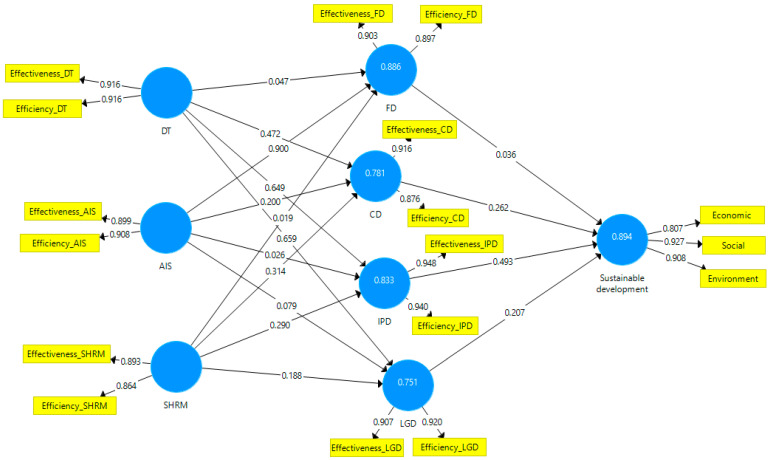
Empirical model. Source: own construction using SmartPLS v3.0 (SmartPLS GmbH, Oststeinbek, Germany).

The SmartPLS v3.0 software (SmartPLS GmbH, Oststeinbek, Germany) is the best solution for testing the four hypotheses because it allows structural equation modeling. The model applied is reflective and uses a PLS algorithm. For path coefficient a bootstrapping procedure is applied to the model. [108]. Figure 3 illustrates the empirical model.

The variables’ reliability and validity are excellent (Table 3), with Cronbach’s Alpha over 0.8, Composite Reliability over 0.8, and average variance extracted over 0.6 [108]. Moreover, values below 0.08 (0.074) for SRMR (standardized root mean squared residual) and values over 0.9 (0.904) for NFI (normed fit index) prove a good fit for the model.

The paper used a bootstrapping procedure (with 500 subsamples and a significance level of 0.05) to test research hypotheses. Values above 2.6 for T statistics and below 0.005 for *p* values show an increased relevance of the path coefficients [108]. The path coefficients indicate direct positive influences among model variables (Table 4).

Table 4 highlights that all four hypotheses are validated. The DT, the improvement of the AIS, and the SHRM have a significant positive effect on the BSC dimensions (Hypotheses H1, H2, and H3). On the other hand, BSC dimensions have a significant positive effect on sustainable development (Hypothesis H4). However, the model also shows some weak influences between the researched variables. Digital transformation and strategic human resource management do not significantly influence the financial dimension of performance. The accounting information system is essential in healthcare managers’ perception of the financial dimension. Human results and technologies are tools that support the accounting information system but do not significantly influence the financial dimension. In turn, the financial dimension of performance does not significantly influence sustainable development since, in the perception of healthcare managers, the essential drivers of sustainability are social and environmental. Regarding the influences exerted by the accounting information system on the BSC performance dimensions, only the influence on the internal process dimension is irrelevant because healthcare managers believe that the accounting information system uses financial information that does not match the operational indicators of the internal process dimension.

## 5. Discussions

The rapid pace of economic changes in recent decades has imposed the need to understand the influences of changes on an organization’s activities and how they can increase performance [109]. In this dynamic and ever-changing environment, knowledge-based assets create most of the organization’s added value, being largely intangible. The intangibility of assets has affected the traditional methods of evaluating organizational performance. As a result, organizations have, since the 1990s, to implement multidimensional valuation models that also consider intangible assets. These models enable the alignment of cost and performance evaluation systems with the organization’s strategy. BSC is a widely used performance evaluation system [9] in various forms.

The proposed theoretical model emphasizes the need to consider the significance of information resources and human resources on the BSC dimensions (FD, CD, IPD, and LGD) and in ensuring organizational sustainability. As per [16], the paper considers that organizations in the healthcare sector that want to achieve sustainability goals must implement innovative solutions within information systems and motivate their employees to use the new opportunities offered by digitalization [110].

Implementing new digital technologies, improving accounting information systems, and optimizing strategic human resources management influence the BSC performance dimensions (Hypotheses H1, H2, H3). The study results show that digital transformation mainly influences internal processes and learning and growth dimensions. Furthermore, improving accounting information systems exerts significant influences, especially on the financial and customer dimensions. On the other hand, optimizing strategic human resources management significantly affects the internal process and customer dimensions.

Similar to the findings of Kilig and Uludag [105], Gazi et al. [9], and Alnamrouti et al. [31], the paper’s results show that organizations aimed to improve performance must adapt to informational and DT by motivating employees to develop their knowledge and skills (Hypotheses H1, H2, H3). Like Fabac [27], the paper demonstrates that implementing new digital technologies and innovations in an organization represents a significant competitive advantage. In line with the findings of Gazi et al. [9] and Dudic [75], improving the accounting information systems of organizations in the healthcare sector through digital transformation and the strategic change in human resources has a significant impact on aligning the organizational strategy with the objectives of increasing performance and sustainable development. The investigation of hypothesis H4 led to the conclusion that the BSC dimensions have a significant positive effect on sustainable development, with the internal process dimension exerting the most decisive influence on the organization’s sustainable development. Similar to Fabac [23], the research results show that digitalization and sustainability are the dominant global social phenomena in the contemporary period, and strategic management cannot ignore the effects of digitalization on performance evaluation through the BSC and, finally, on sustainable development. Therefore, the organizational transformation achieved by considering the effects of implementing new digital technologies, improving accounting information systems, and strategic human resources management must also include sustainable development objectives.

### 5.1. Practical and Managerial Implications

Organizations in the healthcare sector are at various stages of BSC implementation as an organizational performance evaluation system. This study aimed to determine the influences of DT, the improvement of the AIS, the SHRM on BSC, and the indirect impact on sustainable development in the perception of employees with management experience in the healthcare sector. The study results enable managers in the healthcare sector to understand the role of information and digital systems and SHRM in implementing BSC to achieve sustainable development. Furthermore, based on the study’s results, managers in the healthcare sector can implement or restructure the existing performance evaluation system to align with the organization’s strategic objectives following the imperatives of the technological revolution 4.0 and sustainable development.

Therefore, healthcare organizations aiming for sustainable development must implement BSC, relying on digital transformation, improvement of the accounting information system, and a strategic approach to human resources to obtain their commitment to the implementation of the organizational strategy. The central policies and actions that healthcare managers must promote are:

- encouraging the integration of digital technologies in all medical or auxiliary activities of the organization;
- the restructuring of the accounting information systems in the BSC-sized functions and the relevant management policies;
- promotion of a more dynamic, inclusive, and performance-oriented environment among employees;
- promoting a leadership based on innovation and creativity;
- encouraging engagement in achieving sustainability objectives by obtaining employee loyalty;
- supporting employee confidence and self-development, encouraging autonomy, and allowing participation in decisions.

### 5.2. Theoretical Implications

Over the past three years, healthcare systems have developed resilience to deal with the COVID-19 pandemic [19], manage resources optimally, and align their goals with the mission of providing efficient and effective healthcare [111]. In addition, as a result of multiple public sector reforms [112], healthcare systems have developed performance measurement systems to improve the efficiency and effectiveness of healthcare [19,113,114,115,116,117].

The sustainable development of organizations depends on how managers understand how to ensure increased efficiency and effectiveness by aligning performance objectives with organizational strategies. In increasing the efficiency and effectiveness of contemporary organizations, an important role plays DT, the optimization of AIS, and the SHRM. This study investigated the relationship between digital transformation, the accounting information system, strategic human resources management, the BSC dimensions, and the sustainable development of organizations, offering a perspective on integrating these variables into the organization’s strategy. The positive influences found between the research variables indicate the need to implement new digital technologies to improve accounting information systems and the motivation of employees to use these new technologies to increase performance. The proposed model provides the tools to integrate the BSC with the HTA, analyzing the relationships between digital transformation, accounting information systems, strategic human resources management, and BSC dimensions. Through the proposed model, the paper offers an instrumental framework for BSC implementation in line with digital transformation and the drivers of sustainable development.

### 5.3. Limitations and Further Research

Like other investigations, this research has limitations. The first limitation refers to the synthetic approach without describing the indicators of each BSC dimension. Including analytical indicators would provide information on their relevance within each dimension in the perception of employees with management experience in the healthcare sector. Future research should explore the influence of BSC dimensions on each vector of sustainability (economic, social, and environmental) to provide management with clues as to which performance dimensions to focus on in achieving sustainable development. In addition, other factors can influence the optimal implementation of BSC. A second limitation is the construction of the sample (employees with management experience in the healthcare sector in Romania), which does not provide increased representativeness. Extending research to other countries will allow researchers to consider organizational culture as an influencing factor of BSC dimensions, providing a clearer picture of employees’ perceptions.

## 6. Conclusions

The challenges generated by globalization, competitiveness, and the permanent requirement for performance improvement led modern organizations to find new ways to increase performance sustainably. The digital transformation of accounting information systems and the development of human resources skills to use new digital tools is an optimal way to achieve the sustainable development of organizations in the healthcare sector, focused on a knowledge economy.

The research suggests that organizations should consider the effects of digital transformation and employee acceptance on organizational performance to meet sustainable development challenges. The study emphasized the influence factors on the performance dimensions within the BSC and the effects of financial and non-financial performance on sustainable development. The study concludes that implementing the BSC is particularly useful for ensuring sustainable development, regardless of its challenges. If the support provided by organizational managers for digital transformation is not convincing, the overall effort will fail, causing low performance in conditions of unsustainable development.

## Figures and Tables

**Table 1:** Questionnaire design.

Variables	Items	Scales
Demographic variables	Gender	Male (1), Female (2)
	Age	18–30 years (1), 31–45 years (2), 46–65 years (3)
DT	Efficiency_DT	1 to 5 (1—non-important, 5—most important)
	Effectiveness_DT	
AIS	Efficiency_AIS	
	Effectiveness_AIS	
SHRM	Efficiency_SHRM	
	Effectiveness_ SHRM	
FD	Efficiency_FD	
	Effectiveness_FD	
CD	Efficiency_CD	
	Effectiveness_CD	
IPD	Efficiency_IPD	
	Effectiveness_IPD	
LGD	Efficiency_LGD	
	Effectiveness_LGD	
Sustainable development	Economic	
	Social	
	Environment	

Source: own construction based on [7,9,31,42,106,107].

**Table 2:** Descriptive statistics.

	Min	Max	Mean	Std. Deviation	Skewness	Kurtosis
Sex	1	2	1.46	0.499	0.172	−1.981
Age	1	3	2.07	0.752	−0.111	−1.221
Efficiency_DT	1	5	3.79	1.004	−0.470	−0.575
Effectiveness_DT	1	5	3.65	1.058	−0.455	−0.422
Efficiency_AIS	2	5	4.01	0.943	−0.548	−0.731
Effectiveness_AIS	2	5	3.83	0.995	−0.388	−0.925
Efficiency_SHRM	2	5	3.95	0.880	−0.472	−0.525
Effectiveness_SHRM	1	5	3.86	0.947	−0.563	−0.297
Efficiency_FD	1	5	3.84	0.972	−0.434	−0.722
Effectiveness_FD	2	5	4.02	0.947	−0.594	−0.660
Efficiency_CD	2	5	3.97	0.952	−0.492	−0.806
Effectiveness_CD	1	5	3.80	0.959	−0.450	−0.405
Efficiency_IPD	1	5	3.61	0.974	−0.209	−0.649
Effectiveness_IPD	2	5	3.87	0.934	−0.361	−0.806
Efficiency_LGD	1	5	3.59	0.952	−0.120	−0.825
Effectiveness_LGD	1	5	3.83	0.869	−0.467	−0.250
Economic	1	5	3.81	0.856	−0.531	0.029
Social	2	5	3.83	0.927	−0.297	−0.824
Environment	1	5	3.42	1.211	−0.316	−0.810

Source: own construction using SPSS v.20 (SPSS Inc., Chicago, IL, USA).

**Table 3:** Validity and reliability.

	Cronbach’s Alpha	Composite Reliability	AVE
AIS	0.775	0.899	0.816
CD	0.758	0.891	0.804
DT	0.808	0.913	0.839
FD	0.766	0.895	0.810
IPD	0.878	0.942	0.891
LGD	0.802	0.910	0.835
SHRM	0.705	0.871	0.772
Sustainable development	0.856	0.913	0.778

Source: own construction using SmartPLS v3.0 (SmartPLS GmbH, Oststeinbek, Germany).

**Table 4:** Path coefficients.

Hypotheses	Path	Original Sample	T Statistics	*p* Values	Validation
H1	DT → FD	0.047	1.581	0.114	Validated
	DT → CD	0.472	11.547	0.000	
	DT → IPD	0.649	17.390	0.000	
	DT → LGD	0.659	14.717	0.000	
H2	AIS → FD	0.900	45.083	0.000	Validated
	AIS → CD	0.200	5.177	0.000	
	AIS → IPD	0.026	1.002	0.317	
	AIS → LGD	0.079	2.180	0.030	
H3	SHRM → FD	0.019	0.732	0.465	Validated
	SHRM → CD	0.314	8.185	0.000	
	SHRM → IPD	0.290	7.696	0.000	
	SHRM → LGD	0.188	4.339	0.000	
H4	FD → Sustainable development	0.036	1.398	0.163	Validated
	CD → Sustainable development	0.262	6.966	0.000	
	IPD → Sustainable development	0.493	10.589	0.000	
	LGD → Sustainable development	0.207	4.764	0.000	

Source: own construction using SmartPLS v3.0 (SmartPLS GmbH, Oststeinbek, Germany).

## Data Availability

Not applicable.

## Appendix A

**Table A1:** Questionnaire items.

Variables	Items
Demographic variables	What is your gender?
	What is your age range?
DT	On a scale from 1 to 5 (1—non-important, 5—most important), what do you think is the importance of DT/in increasing organizational efficiency?
	On a scale from 1 to 5 (1—non-important, 5—most important), what do you think is the importance of DT in increasing organizational effectiveness?
AIS	On a scale from 1 to 5 (1—non-important, 5—most important), what do you think is the importance of AIS in increasing organizational efficiency?
	On a scale from 1 to 5 (1—non-important, 5—most important), what do you think is the importance of AIS in increasing organizational effectiveness?
SHRM	On a scale from 1 to 5 (1—non-important, 5—most important), what do you think is the importance of SHRM in increasing organizational efficiency?
	On a scale from 1 to 5 (1—non-important, 5—most important), what do you think is the importance of SHRM in increasing organizational effectiveness?
FD	On a scale from 1 to 5 (1—non-important, 5—most important), what do you think is the importance of BSC’s FD in increasing organizational efficiency?
	On a scale from 1 to 5 (1—non-important, 5—most important), what do you think is the importance of BSC’s FD in increasing organizational effectiveness?
CD	On a scale from 1 to 5 (1—non-important, 5—most important), what do you think is the importance of BSC’s CD in increasing organizational efficiency?
	On a scale from 1 to 5 (1—non-important, 5—most important), what do you think is the importance of BSC’s CD in increasing organizational effectiveness?
IPD	On a scale from 1 to 5 (1—non-important, 5—most important), what do you think is the importance of BSC’s IPD in increasing organizational efficiency?
	On a scale from 1 to 5 (1—non-important, 5—most important), what do you think is the importance of BSC’s IPD in increasing organizational effectiveness?
LGD	On a scale from 1 to 5 (1—non-important, 5—most important), what do you think is the importance of BSC’s LGD in increasing organizational efficiency?
	On a scale from 1 to 5 (1—non-important, 5—most important), what do you think is the importance of BSC’s LGD in increasing organizational effectiveness?
Sustainable development	On a scale of 1 to 5 (1—non-important, 5—most important), what do you think is the importance of economic drivers in ensuring sustainable development
	On a scale of 1 to 5 (1—non-important, 5—most important), what do you think is the importance of social drivers in ensuring sustainable development
	On a scale of 1 to 5 (1—non-important, 5—most important), what do you think is the importance of environmental drivers in ensuring sustainable development

Source: own construction based on [7,9,31,42,106,107].
